# Role of CD68 in tumor immunity and prognosis prediction in pan-cancer

**DOI:** 10.1038/s41598-022-11503-2

**Published:** 2022-05-12

**Authors:** Jingwei Zhang, Shuwang Li, Fangkun Liu, Kui Yang

**Affiliations:** Department of Neurosurgery, Xiangya Hospital, Central South University, Changsha, 410008 People’s Republic of China

**Keywords:** Cancer, Drug discovery, Genetics, Immunology, Biomarkers, Neurology, Risk factors

## Abstract

CD68 plays a critical role in promoting phagocytosis; however, the function of CD68 in tumor immunity and prognosis remains unknown. We analyzed CD68 expression among 33 tumor and normal tissues from The Cancer Genome Atlas and Genotype-Tissue Expression datasets. The relationship between CD68 expression and cancer prognosis, immune infiltration, checkpoint markers, and drug response was explored. Upregulated CD68 levels were observed in various cancer types, which were verified through tumor tissue chips using immunohistochemistry. High levels of CD68 in tumor samples correlated with an adverse prognosis in glioblastoma, kidney renal clear cell carcinoma, lower-grade glioma, liver hepatocellular carcinoma, lung squamous cell carcinoma, thyroid carcinoma, and thymoma and a favorable prognosis in kidney chromophobe. The top three negatively enriched Kyoto Encyclopedia of Genes and Genomes terms in the high CD68 subgroup were chemokine signaling pathway, cytokine-cytokine receptor interaction, and cell adhesion molecule cams. The top negatively enriched HALLMARK terms included complement, allograft rejection, and inflammatory response. A series of targeted drugs and small-molecule drugs with promising therapeutic effects were predicted. The clinical prognosis and immune infiltration of high expression levels of CD68 differ across tumor types. Inhibiting CD68-dependent signaling could be a promising therapeutic strategy for immunotherapy in many tumor types.

## Introduction

According to a recent study in 2019, cancer has become the leading or second most common cause of death in more than 112 countries in people less aged than 70 years^1^. Worldwide, more than 19 million new cancer cases and 10 million cancer-related deaths occurred in 2020^2^. Despite undergoing a series of traditional treatment methods, including radiotherapy, chemotherapy, biological therapy, and surgery, the effect of tumor therapy remains unsatisfactory^3,4^. Tumor immunotherapy strategies, such as focusing on programmed cell death protein 1 (PD-1), were shown to be novel and promising treatments for tumors^5,6^. With the rapid development of high-throughput sequencing technology, an increasing number of immune-associated molecules related to tumor prognosis have been discovered, which may play an irreplaceable role in tumor immunotherapy.

The cluster of differentiation 68 (CD68), also known as GP110, LAMP4, or SCARD1, is a 110 kDa transmembrane glycoprotein that is widely expressed in monocyte cell types, such as macrophages, microglia, and osteoclasts^7^. CD68 plays an essential role in various physiological and pathological processes, including atherosclerosis formation^8^, inflammation and auto-immunity^9^, bone-resorbing promotion^10^, and tumor progression^11,12^. Bone marrow-derived macrophages are the most common type of tumor-infiltrating immune cells in the tumor microenvironment (TME) and are vital mediators of the antitumor immune response^13–15^. Recent studies have found that CD68 is overexpressed in tumor-associated macrophages (TAMs) and tumor cells. High levels of CD68 are associated with a higher tumor grade, larger tumor size, Ki67 positivity, and other malignant features, which indicate tumor progression and aggressiveness^16–19^. TAMS, identified by CD68 expression, can be divided into two subtypes: classically activated type 1(M1-like) macrophages and alternatively activated type 2 (M2-like) macrophages. M1-like macrophages, with proinflammatory characteristics that express high levels of free radicals and major histocompatibility complex molecules, contribute to antitumor activity^20,21^. In contrast, M2-like macrophages, which release multiple anti-inflammatory cytokines and chemokines, promote tumor growth and metastasis^22,23^. Increasing evidence has shown that CD68 is a promising tumor-associated diagnostic and prognostic marker for cancer. However, the signaling pathways in which CD68 is involved in tumor immunity and progression remain unclear.

In this study, we studied the expression of *CD68* in 33 cancer types using large-scale RNA-sequencing (RNA-seq) data from The Cancer Genome Atlas (TCGA). Upregulated levels of CD68 were observed in various cancer types, which were observed in the TCGA database and our tumor tissue chips. We also discussed the value of CD68 in the prognostic prediction of pan-cancer. Moreover, the relationship between the expression levels of *CD68* and the infiltration of immune cells in the pan-cancer microenvironment was observed. Finally, we analyzed the correlation between a series of predicted drugs and *CD68* expression, which may be used for tumor immunotherapy in the future.

## Methods

### Collection of sample and patient data

The clinicopathological features and RNA-sequencing (RNA-seq) data of the 33 types of cancer were chosen from the TCGA dataset (http://cancergenome.nih.gov). As the data from normal tissue is relatively insufficient, the RNA-seq data of normal human tissues were additionally added from the GTEx dataset (https://www.gtexportal.org/) to analyze the expression levels of *CD68* between tumor and normal tissues. A tumor tissue chip (catalog No. BCN963) contains multiple organ tumor arrays with matched normal tissues that were used to verify the expression of CD68. Informed consent was obtained from all participants in this study.

### Recognition of relevant features

The gene expression data of *CD68* were extracted from the TCGA and GTEx databases to form an expression matrix using GEPIA (http://gepia.cancer-pku.cn/) and R package (4.0.4). The genetic mutation aspects of *CD68* were obtained from the public database CBIOPORTAL (https://www.cbioportal.org/). A Kaplan–Meier (KM) analysis with a log-rank test was used to compare the disease-free interval (DFI), progression-free interval (PFI), disease-specific survival (DSS), and overall survival (OS) of the patients. In this study, we selected the optimal cutoff point to create KM curves based on the coxph function of R package survival. A univariate Cox model was used to calculate the relationship between *CD68* expression levels and patient survival. The immune infiltrates among 33 types of cancers were studied using the Tumor Immune Estimation Resource (TIMER 2.0, https://cistrome.shinyapps.io/timer/)^24^ and CIBERSORT^25^. The ESTIMATE algorithm was applied to estimate the stromal and immune cells in the tumor microenvironment and to calculate the stromal and immune scores and estimate scores. Gene set enrichment analysis (GSEA) was used to display the biological functions and pathways involved in *CD68*. This analysis was implemented in Sangerbox (http://sangerbox.com/) based on the Molecular Signatures Database (MSigDB) H (hallmark gene sets) and Kyoto Encyclopedia of Genes and Genomes database (KEGG)^26–28^. The relationship between CD68 expression and drug responses was predicted using CELLMINER (http://discover.nci.nih.gov/cellminer/) using the R language. In addition, sensitive drugs based on *CD68* levels were analyzed from the Clinical Trials Reporting Program and Genomics of Drug Sensitivity in Cancer database.

### Immunohistochemistry

In this study, the immunohistochemistry protocol was used to stain the tissue chip BCN963 (Bioyeargene Biosciences company, Wuhan, China) as previously reported^29^. Briefly, sections were obtained from formalin-fixed, paraffin-embedded tissues of normal and pan-cancer. After antigen retrieval and blocking of endogenous HRP activity, the slides were blocked with 10% normal goat serum and incubated with a primary antibody at 4 °C overnight. Then, the secondary antibody was added and incubated at room temperature for 50 min. Slides were counterstained with hematoxylin, and representative images were obtained using an Olympus inverted microscope. The primary antibodies used were polyclonal rat anti-CD68 (AF20022, AiFang Biological, China, 1:200 dilution). HRP-labeled goat anti-rabbit IgG (GB23303, ServiceBio, 1:200 dilution) was used as the secondary antibody. We optimized the final concentration through a pre-experiment and the protocol of the antibody. Negative control procedures included the omission of the primary antibody.

### Statistical analysis

A student’s t-test was performed to explore the correlation between *CD68* expression and the drugs. The Kruskal–Wallis test was used to compare the expression levels of *CD68* in tumor and normal tissues. KM curves, the log-rank test, and the Cox proportional hazards regression model were applied to analyze the survival conditions. In addition, Spearman’s test was used for the correlation analysis. All analyses were performed using the R language. All statistical tests were two-sided, and statistical significance was set at a P-value of < 0.05.

### Ethical approval and ethical standards

The study with primary human tissues was approved by the ethics committee of the Xiangya Hospital, Central South University, and the procedures with human samples were performed in accordance with the ethical standards of the ethics committee and the Helsinki Declaration of 1975 and its later amendments.

## Results

### Expression of CD68 in pan-cancer

**Figure 1:**
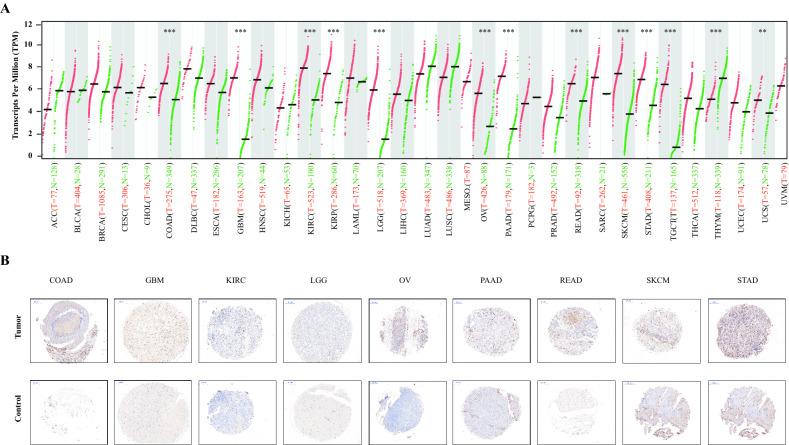
Expresssion aspect of CD68 in tumor and normal tissues based on the TCGA and GETx databases. *CD68* expression from the TCGA and GTEx databases (**A**). CD68 expression from tumor tissue chip using immunohistochemistry, n = 3 for tumor/control groups in COAD, GBM, KIRC, LGG, OV, PAAD, READ, SKCM, and STAD (**B**). *P < 0.05, **P < 0.01, ***P < 0.001.

First, to fully clarify the expression of *CD68* in pan-cancer, we matched the GTEx normal samples with TCGA tumor samples (Fig. 1A). We found that the levels of *CD68* were significantly elevated (P < 0.01) in colon adenocarcinoma (COAD), glioblastoma multiforme (GBM), kidney renal clear cell carcinoma (KIRC), kidney renal papillary cell carcinoma (KIRP), brain low-grade glioma (LGG), ovarian serous cystadenocarcinoma (OV), pancreatic adenocarcinoma (PAAD), rectum adenocarcinoma (READ), skin cutaneous melanoma (SKCM), stomach adenocarcinoma (STAD), testicular germ cell tumors (TGCT), and uterine carcinosarcoma (UCS) compared to in normal tissues. In contrast, *CD68* was significantly decreased (P < 0.001) in thymoma (THYM) compared with GTEx normal controls. Moreover, we observed the expression of CD68 in these tumor and normal controls using tissue chip. Each tumor/normal tissue has three cores (diameter 1 mm). Even though statistical analysis was not possible due to the small sample size, we compared the expression of CD68 in these tumor tissues and normal tissues and found that the immunohistoactivity of CD68 was obviously enhanced in COAD, GBM, KIRC, LGG, OV, PAAD, READ, SKCM, and STAD compared with their normal controls (Fig. 1B), which might provide evidence for the difference in CD68 gene expression in Fig. 1A.

### Mutation profile and prognostic value of *CD68* in pan-cancer

**Figure 2:**
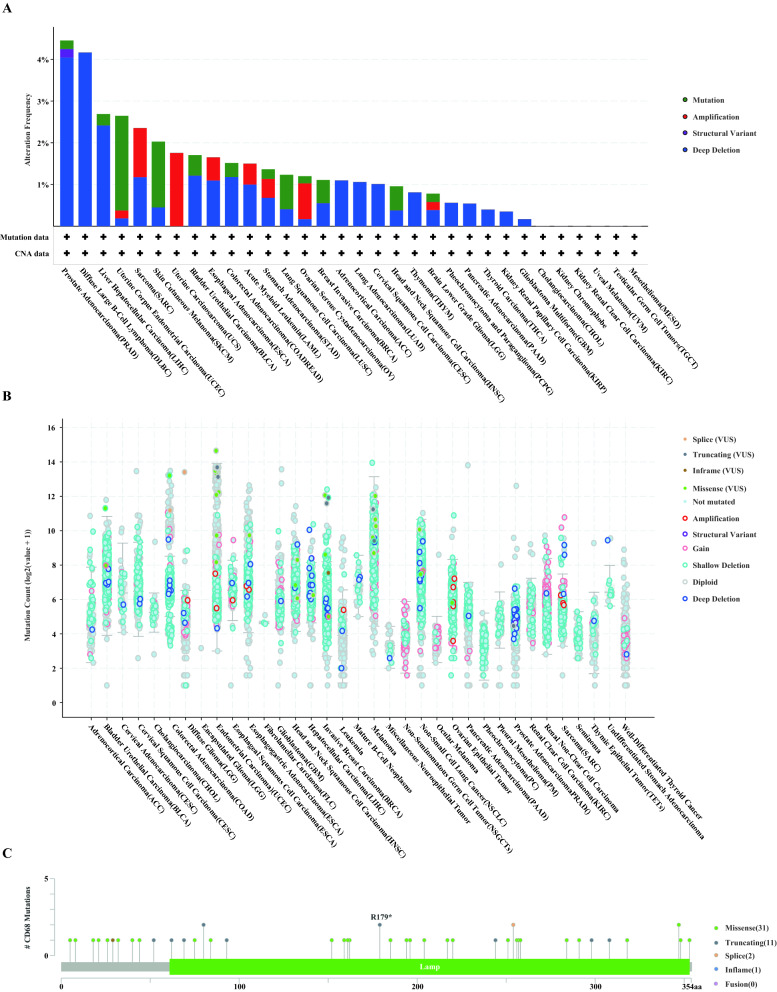
Mutant landscapes of *CD68* in pan-cancer. Mutant frequency (**A**) and count (**B**) of *CD68* in pan-cancer from the TGCA database based on cBioPortal analysis. Mutation aspect of *CD68* in pan-cancer across protein domains (**C**).

**Figure 3:**
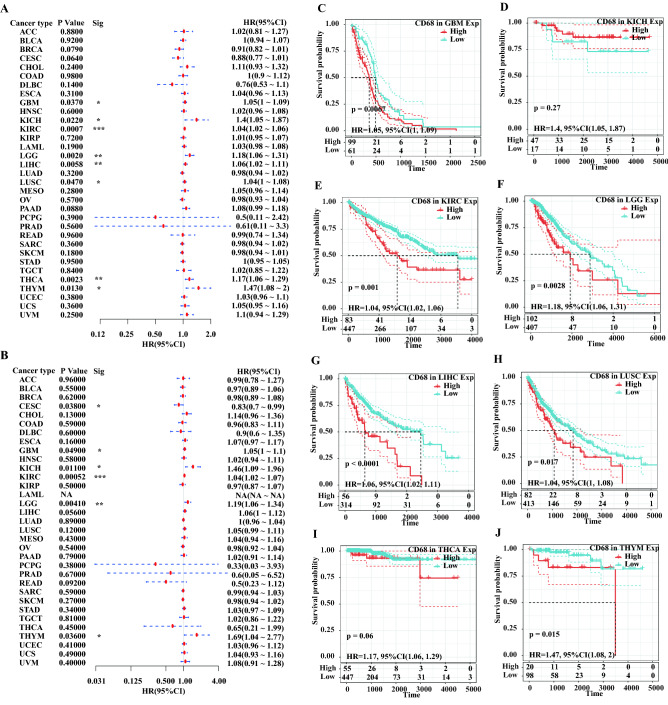
Survival analysis of *CD68* in pan-cancer from the TCGA database. Forest plot compared the prognostic value of *CD68* on OS (**A**) and DSS (**B**) across pan-cancer. Kaplan–Meier Method displayed the prognostic value of *CD68* on OS in GBM (**C**), KICH (**D**), KIRC (**E**), LGG (**F**), LIHC (**G**), LUSC (**H**), THCA (**I**), THYM (**J**). The cut-off points are 61.89% (GBM), 73.44% (KICH), 15.66% (KIRC), 20.04% (LGG), 15.14% (LIHC), 16.57% (LUSC), 10.96% (THCA), 16.95% (THYM). *P < 0.05, **P < 0.01, ***P < 0.001.

We then checked the mutant landscape of *CD68* in different cancer types from the TCGA database using cBioportal (Fig. 2). The data showed that prostate adenocarcinoma (PRAD) and diffuse large B-cell lymphoma had a high mutation level with *CD68* deep deletion of more than 4% (Fig. 2A,B). A total of 45 mutation sites (including 31 missense, 11 truncating, two splices, and one inflame) were found between amino acids 0 and 354 (Fig. 2C). Next, to understand the prognostic value of *CD68* in pan-cancer further, we downloaded RNA-seq and clinical data of *CD68* from the TCGA dataset. Elevated levels of CD68 were significantly related to poorer OS in GBM (hazard ratio [HR] 1.05, 95% confidence interval [CI] 1.00–1.09, P = 0.0370), KIRC (HR 1.04, 95% CI 1.0–21.06, P = 0.0007), LGG (HR 1.18, 95% CI 1.06–1.31, P = 0.0020), liver hepatocellular carcinoma (LIHC) (HR 1.06, 95% CI 1.02–1.11, P = 0.0058), lung squamous cell carcinoma (LUSC) (HR 1.04, 95% CI 1.00–1.08, P = 0.0470), thyroid carcinoma (THCA) (HR 1.17, 95% CI 1.06–1.29, P = 0.0023), and thymoma (THYM) (HR 1.47, 95% CI 1.08–2.00, P = 0.0130) (Fig. 3). In contrast, upregulated *CD68* expression was associated with a favorable prognosis in kidney chromophobe (KICH) (HR 1.40, 95% CI 1.05–1.87, P = 0.0220). In addition, the high expression of *CD68* was related to an unfavorable DSS in KIRC (HR 1.04, 95% CI 1.02–1.07, P = 0.0052), GBM (HR 1.05, 95% CI 1.00–1.10, P = 0.0490), LGG (HR 1.19, 95% CI 1.06–1.34, P = 0.0410), and THYM (HR 1.69, 95% CI 1.04–2.77, P = 0.0360) (Fig. 3B and Supplementary Fig. 1C–I). However, DSS was favorable in cervical squamous cell carcinoma and endocervical adenocarcinoma (CESC) (HR 0.83, 95% CI 0.70–0.99, P = 0.0380) and KICH (HR 1.46, 95% CI 1.09–1.96, P = 0.0110). Furthermore, we observed the prognostic value of *CD68* in DFI (Supplementary Fig. 1A) and PFI (Supplementary Fig. 1B). The results showed that high levels of *CD68* were associated with a poorer DFI in GBM, LGG, and THYM and a better DFI in KICH (Supplementary Fig. 1E–I). High levels of *CD68* were associated with a poorer PFI in cholangiocarcinoma (CHOL), LIHC, and STAD and a better DFI in CESC (Supplementary Fig. 1J–M).

### Relationship between *CD68* expression and immune infiltrates

**Figure 4:**
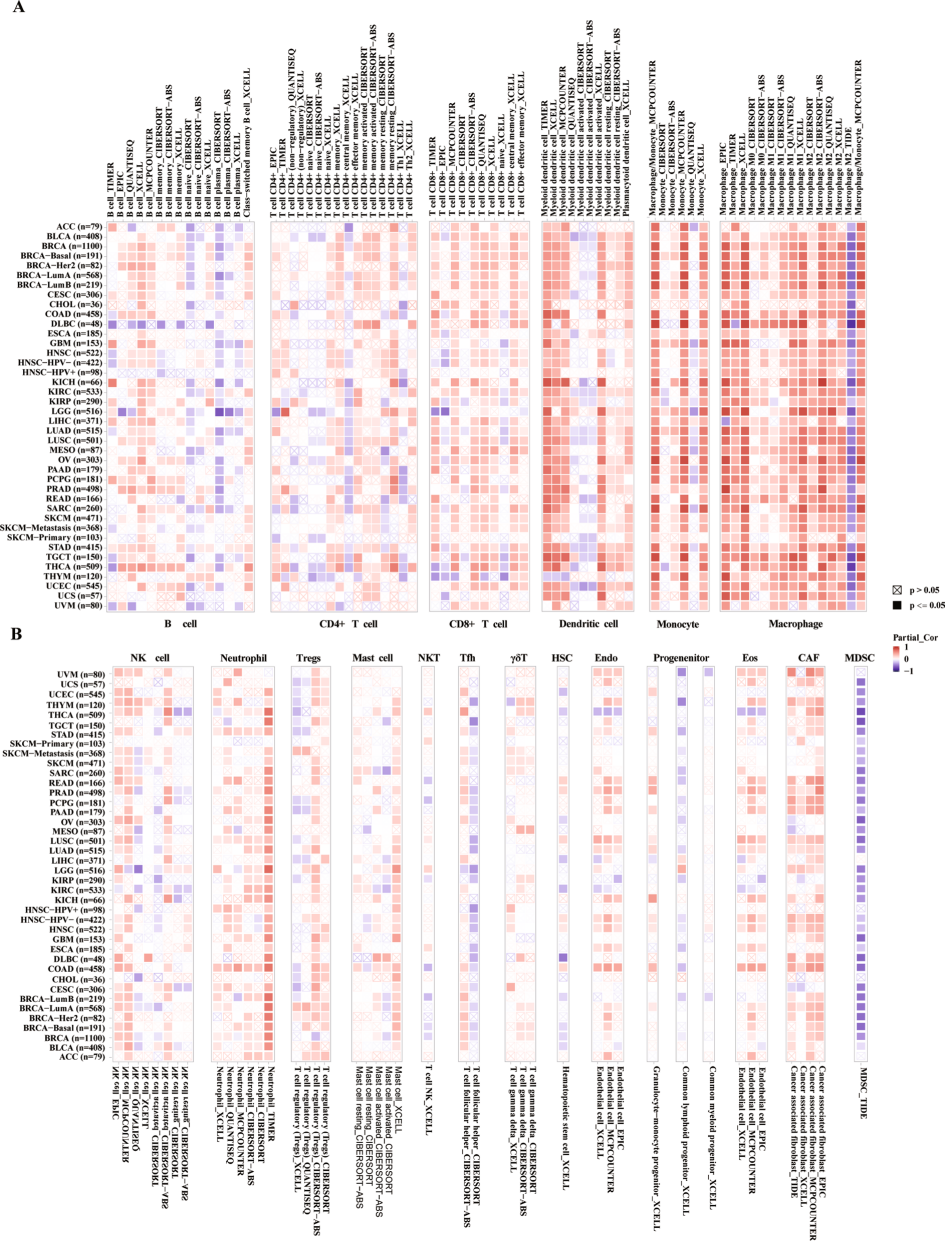
The relationship between *CD68* and immune infiltrates based on the TIMER2.0 analysis in pan-cancer. The correlation between *CD68* with B cell, CD4^+^ T cell, CD8^+^ T cell, dendritic cell, monocyte, macrophage in pan-cancer (**A**). The correlation between *CD68* with NK cell, neutrophil, Tregs, mast cell, NKT, Tfh, γδT, HSC, Endo, progenitor, Eos, CAF, and MDSC in pan-cancer (**B**).

**Figure 5:**
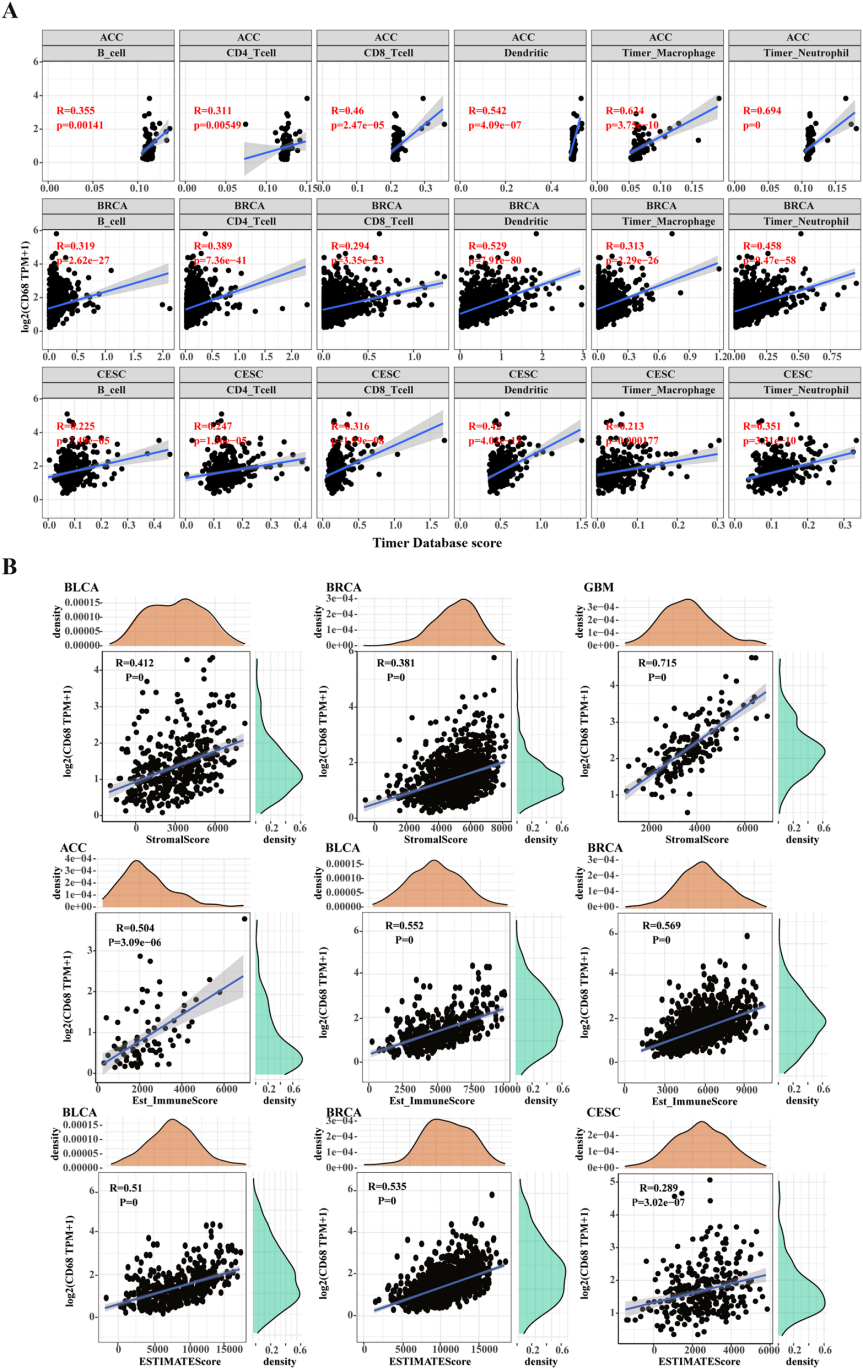
Correlation of *CD68* expression with immune infiltrates based on the CIBERSORT analysis in pan-cancer. Top three cancer types that most related to immune cell infiltration (**A**) and stromal score, immune score, and estimate score (**B**) in pan-cancer.

Next, we explored the relationship between *CD68* expression and immune infiltrates in the tumor microenvironment in 33 tumor types based on the TIMER2.0 database. As shown in Fig. 4, *CD68* expression was positively correlated with immune cell infiltration, including dendritic cells, monocytes, macrophages, and neutrophils. However, *CD68* expression is negatively associated with the infiltration of myeloid-derived suppressor cells. Next, we analyzed the correlation between *CD68* levels and immune cell infiltration in the tumor microenvironment in 33 cancer types. The results indicated that the expression of *CD68* was positively related to the abundance of B cells, CD4^+^ and CD8^+^ T cells, dendritic cells, macrophages, and neutrophils in many tumor types. As shown in Fig. 5A, the three most significantly related tumors were adrenocortical carcinoma (ACC), BRCA, and CESC. The details of other tumor types are shown in Supplementary Fig. 2. We calculated the stromal, immune, and estimated score of 33 cancer types using the ESTIMATE algorithm. As shown in Fig. 5B, the top three tumor types with *CD68* expression positively correlated with stromal score were BLCA, BRCA, and GBM (P < 0.001). The top three tumor types with *CD68* expression positively correlated with immune score were ACC, BLCA, and BRCA (p < 0.001). The top three tumor types with *CD68* expression positively correlated with the estimate score were BLCA, BRCA, and CESC (P < 0.001). The data in Supplementary Fig. 3 shows that the expression of *CD68* was significantly and positively correlated with the stromal score in all tumor types except CHOL and mesothelioma (MESO). In addition, *CD68* levels were found to significantly and positively correlate with the immune score (Supplementary Fig. 4) and estimate the score (Supplementary Fig. 5) in all tumor types. These results indicate that *CD68* has a close relationship with immune infiltrates in the tumor microenvironment and might act as a promising immunotherapy target.

**Figure 6:**
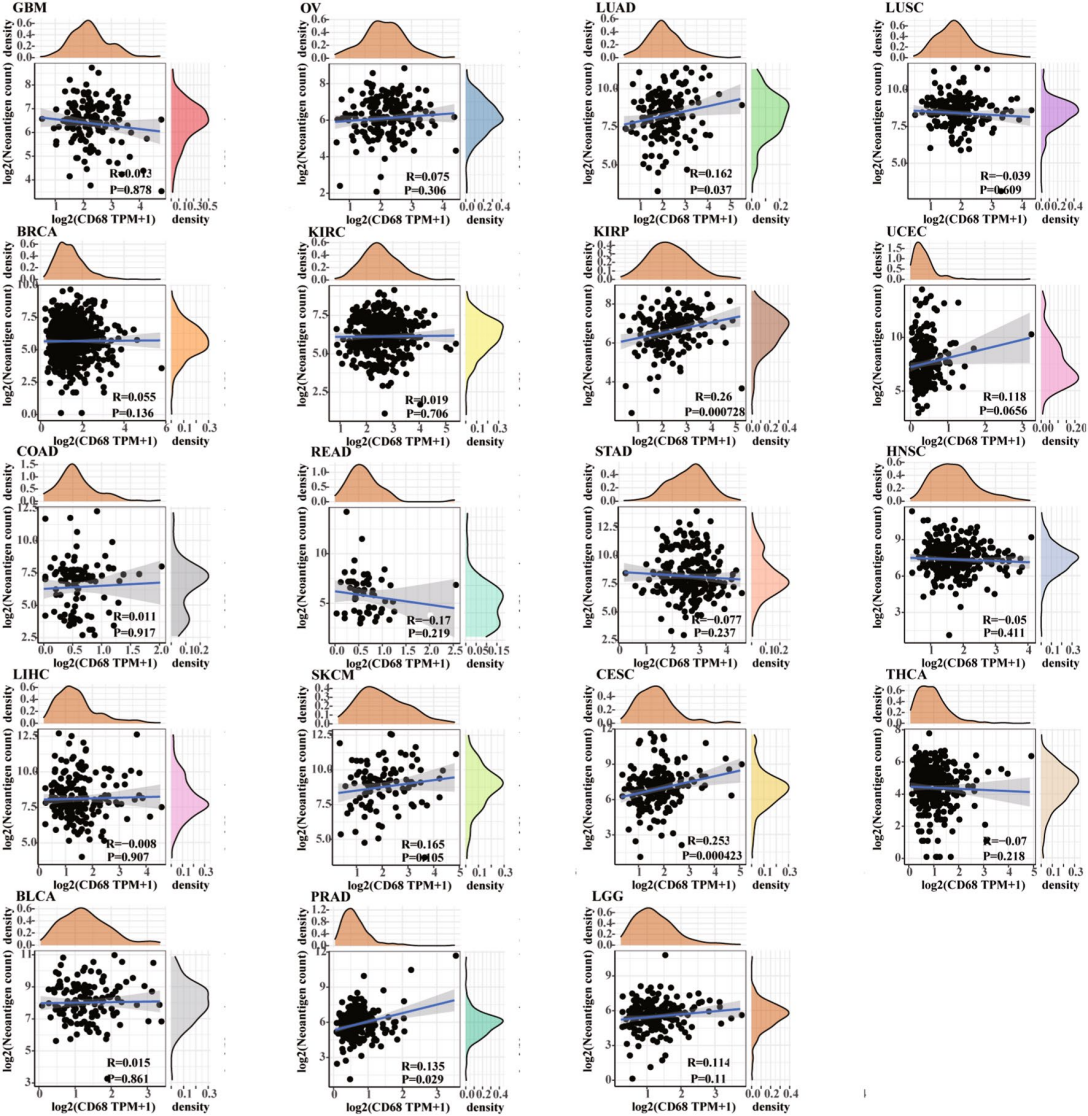
Relationship between neoantigen counts and *CD68* expression in pan-cancer.

Tumor neoantigens are foreign proteins absent from normal human organs/tissues and are encoded by a mutated gene of tumor cells, which plays a crucial role in tumor immunotherapy. We then explored the relationship between *CD68* expression and the number of neoantigens in human cancers (Fig. 6 and Supplementary Table 1). Our results indicated that high levels of *CD68* were significantly and positively related to the number of neoantigens in LUAD, KIRP, CESC, and PRAD (P < 0.05).

### Relationship between *CD68* expression and checkpoint gene markers, tumor mutation burden, and microsatellite instability

**Figure 7:**
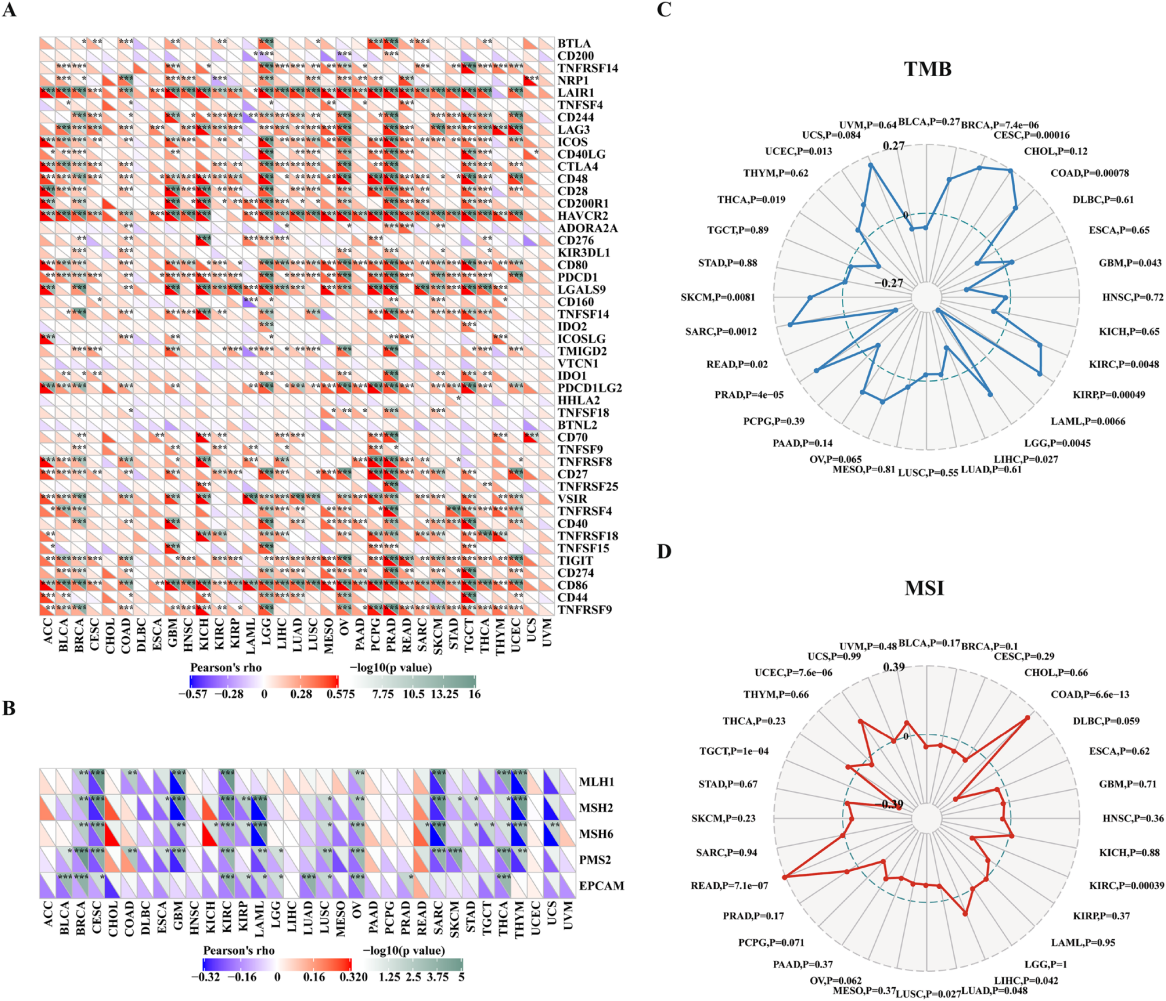
Relationship between immune checkpoints, DNA mismatch repair markers, TMB, MSI, and *CD68* expression in pan-cancer. Correlation of *CD68* expression with various immune checkpoints (**A**), DNA mismatch repair markers (**B**), TMB (**C**) and MSI (**D**) in pan-cancer. *P< 0.05, **P < 0.01, ***P < 0.001.

To further elucidate the potential immune mechanisms of *CD68*, we next compared the association of *CD68* expression with various checkpoint markers in different cancer types (Fig. 7A). The results showed that *CD68* expression positively correlated with the expression of LAIR1, HAVCR2, LGALS9, and PD-1 (PDCD1) in most of the 33 tumor types. We also studied the relationship between *CD68* expression and five DNA mismatch repair (MMR) markers (Fig. 7B). *CD68* levels were significantly and negatively correlated with mutL homolog 1, mutS homolog 2, mutS homolog 6 (MSH6), postmeiotic segregation increased 2 (PMS2), and epithelial cell adhesion molecule in BRCA, CESC, KIRC, OV, and THCA (P < 0.05). However, *CD68* levels were significantly and positively correlated with MSH6 in KICH and READ (P < 0.05). In addition, we studied the correlation between tumor mutation burden (TMB) and microsatellite instability (MSI) with *CD68* levels. Moreover, *CD68* expression was positively correlated (P < 0.05) with TMB in UCEC, SKCM, sarcoma (SARC), READ, PRAD, LGG, KIRP, KIRC, COAD, CESC, and BRCA, and negatively correlated (P < 0.05) with TMB in THCA, READ, LIHC, LAML, and GBM (Fig. 7C). CD68 expression was positively correlated (P < 0.05) with MSI in UCEC, READ, LIHC, and COAD, but negatively correlated (P < 0.05) with MSI in KIRC, LUAD, LUSC, and TGCT (Fig. 7D). In addition, we analyzed the prognostic value of the combination of *CD68* expression and these markers (MMR markers and PD-1). The results indicated that the combination of *CD68* expression and MMR markers had prognostic value in KIRC, LGG, PAAD, and SARC (Supplementary Fig. 6A). The combination of *CD68* expression and PD-1 had prognostic value in KIRC, LGG, SKCM, and THYM (Supplementary Fig. 6B).

### Functional analysis by GSEA and drug response of *CD68*

**Figure 8:**
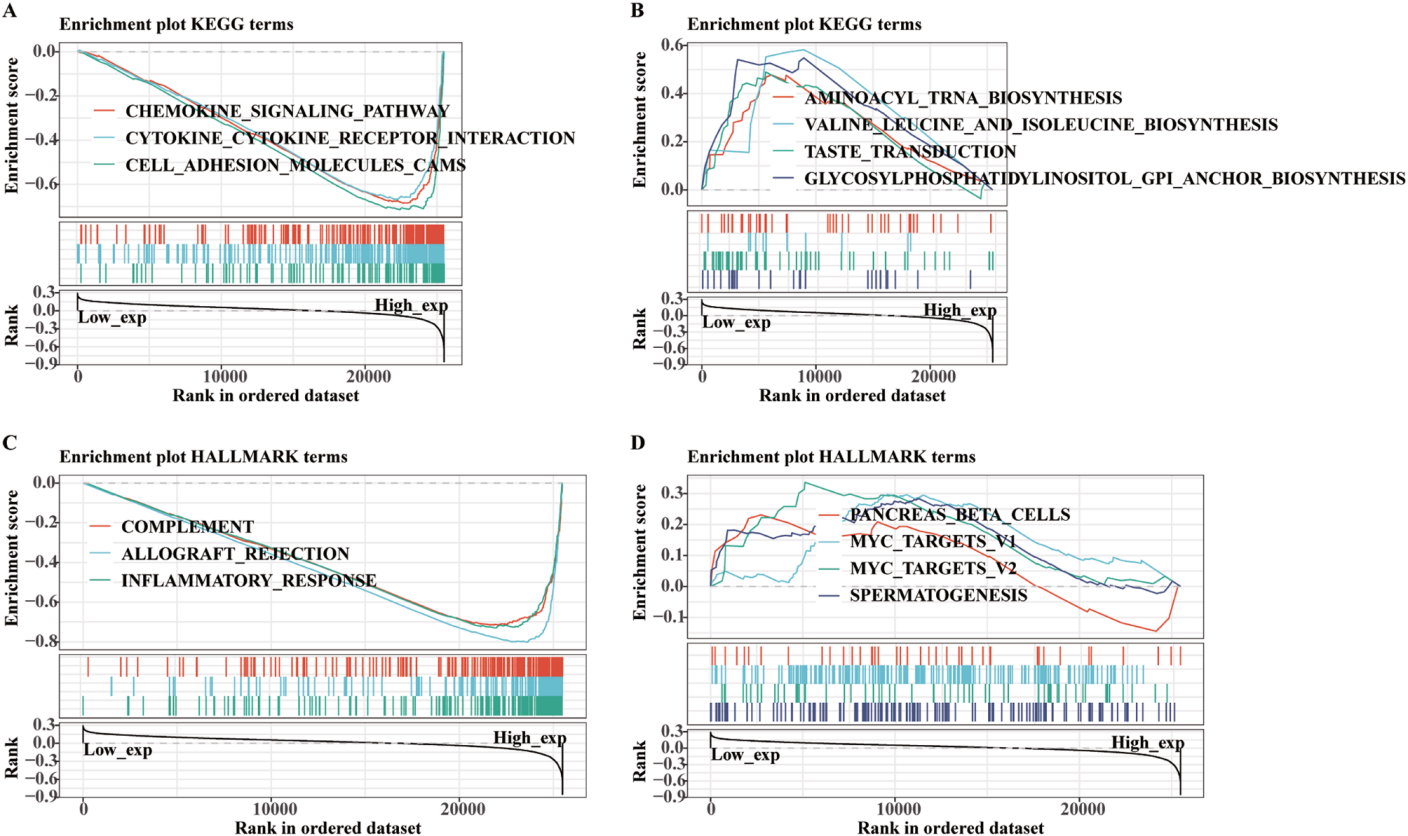
Functional enrichment of KEGG and HALLMARK terms on *CD68* based on GSEA in pan-cancer. The top three negative (**A**) and top four positive (**B**) enriched KEGG terms on *CD68* in pan-cancer. The top three negative (**C**) and top four positive (**D**) enriched HALLMARK terms on *CD68* in pan-cancer.

In addition, we analyzed the related functional signaling pathways of *CD68* through GSEA based on KEGG and HALLMARK databases in pan-cancer. The top three negatively enriched KEGG terms (P < 0.001) in the upregulated *CD68* subgroup were the chemokine signaling pathway, cytokine-cytokine receptor interaction, and cell adhesion molecule cams (Fig. 8A). The top three positively enriched KEGG terms in the upregulated *CD68* subgroup were aminoacyl tRNA biosynthesis, valine leucine, and isoleucine biosynthesis and taste transduction (Fig. 8B). In addition, the top three negatively enriched HALLMARK terms (P < 0.001) in the upregulated *CD68* subgroup were a complement, allograft rejection, and inflammatory response (Fig. 8C). The top three positively enriched HALLMARK terms in the upregulated *CD68* subgroup were pancreatic beta cells and MYC targets V1 and V2 (Fig. 8D). The top five enriched pathways are shown in Supplementary Table 2.

Finally, we analyzed three public databases (CellMiner, CTRP, and Genomics of Drug Sensitivity in Cancer [GDSC]) to identify small molecules and sensitive drugs based on *CD68* expression. The results from drug response analysis by CellMiner suggested that many small molecules were associated with CD68 expression, of which the top 16 are shown in Supplementary Fig. 7. These small molecules were found to be active in inhibiting human tumor cell line growth, including adenocarcinoma, non-small lung, melanoma, prostate, CNS, and colon. In addition, the top 20 sensitive drugs predicted from the CTRP (Supplementary Table 3) and GDSC (Supplementary Table 4) databases exhibited a close relationship with the metabolism and activation of macrophages.

## Discussion

With the rapid development of high-throughput sequencing in recent years, various immune markers in TME that are related to tumor progression have been identified, providing new directions for tumor therapy^30,31^. In this study, we explored the role of *CD68* in clinical outcome prediction and immune cell infiltration in pan-cancer from the TCGA and GETx databases. The results indicated that elevated CD68 was observed in many tumor types, including COAD, GBM, KIRC, KIRP, LGG, OV, PAAD, READ, SKCM, STAD, TGCT, and UCS, and was associated with a more unsatisfactory clinical outcome in GBM, KIRC, LGG, LIHC, LUSC, THCA, and THYM. We also calculated the infiltration of immune cells in the tumor microenvironment. As an important marker of macrophages, CD68 is expressed not only in macrophages, but also in other immune cells and certain tumors^32^. We found that high levels of *CD68* were associated with many immune cells in the tumor microenvironment, such as monocytes, abundant B cells, CD4^+^ and CD8^+^ T cells, dendritic cells, macrophages, and neutrophils. Moreover, the upregulated expression of CD68 was closely related to the stromal, immune, and estimated scores in many types of human cancers. These results were consistent with previous studies in CD68^33–35^, which indicated that *CD68* might be a novel and promising immunotherapy target in the future.

Neoantigens, which are nonautologous proteins with specific characteristics, are generated from the tumor cell genome through nonsynonymous mutations^36^ and play an essential role in tumor immunotherapy^36–38^. The present study illustrated that high expression levels of CD68 were significantly and positively related to the number of neoantigens in LUAD, KIRP, CESC, and PRAD. In addition, the mutation landscape of *CD68* in pan-tumor types was also observed. Anti-immune checkpoint therapy has become a necessary treatment for cancer in recent years^36,39,40^. To fully clarify the immune value of *CD68* in pan-cancer, we next studied the correlation between *CD68* expression and large numbers of immune checkpoints in pan-cancer and found that high levels of *CD68* were significantly and positively correlated with some key checkpoints, including LAIR1, HAVCR2, LGALS9, and PD-1 in most of the 33 tumor types. Furthermore, in this study, *CD68* expression was found to be negatively correlated with DNA mismatch repair (MMR) markers in most types of cancer. Increasing studies have found that TMB and MSI are emerging clinical biomarkers in immunotherapy, clinical outcomes, and chemotherapy sensitivity in various tumor types^41–43^. The present study also found that *CD68* expression was correlated with TMB and MSI in many cancer types, which might provide probable and potential evidence for predicting the efficacy of tumor immunotherapy. The specific mechanisms of *CD68* in tumor growth and metastasis are unclear. Our study explored the majority of pathways related to the expression of *CD68* in pan-cancer, which might help determine the exact function of *CD68* and downstream signaling pathways in the future.

After experiencing many disappointing results in recent years, tumor immunotherapy that focuses on inhibiting immune checkpoint pathways has become a clinically promising treatment for many cancers^44,45^. For example, the PD-1/PD-L1 signaling pathway plays a vital role in the inhibition of immune responses and induction of apoptosis. Several studies have indicated that specific antibodies or drugs that target the PD-1/PD-L1 axis can effectively promote antitumor immunotherapy and lead to a better prognosis in several cancer types^46–48^. Cytotoxic T lymphocyte-associated antigen-4 (CTLA-4), expressed by activated effector T cells, is an important regulator that participates in T cell proliferation and cytokine production^49^. Immunotherapies targeting the CTLA-4 pathway have shown remarkable clinical efficacy against many cancer types, such as prostate, cervical, gastric, pancreatic, ovarian, and urothelial cancers and melanoma^50,51^. In this study, we found that *CD68* is closely related to tumor immunity in the TME and may act as a new immune checkpoint in tumor treatments in the future. To the best of our knowledge, there are no small-molecule drugs that specifically target *CD68* in tumor therapy. Finally, we identified a series of targeted medicines and small-molecule drugs with promising efficacy predicted by *CD68* levels, as proven by the Food and Drug Administration. These sensitive drugs are closely correlated with the proliferation and activation of macrophages. For example, cerulenin, ciclopirox, and PRIMA-1 were the top three predicted drugs that were positively related to *CD68* expression based on the CTRP database. Fatty acid synthase, which induces the synthesis of long-chain fatty acids, prevents proinflammatory progress in macrophages. Cerulenin was found to play a vital role in inhibiting FA synthases in macrophages^52^. Ciclopirox, an iron chelator, can regulate macrophage phagocytosis by depriving iron from macrophages^53^. PRIMA-1, a mutant p53 reactivator, regulates the polarization of macrophages by inhibiting the NF-κB and STAT1 signaling pathways^54^. Moreover, trametinib, RDEA119, and selumetinib were the top three predicted drugs that were negatively related to *CD68* expression based on the GDSC database. Trametinib, RDEA119, and selumetinib, which are effective MEK kinase inhibitors, can significantly reduce the proliferation and migration of macrophages^55,56^. These drugs might play a key role in tumor chemotherapy and are conducive to improving the treatment of tumors.

This study had several limitations. First, the messenger ribonucleic acid expression levels of *CD68* were assessed from public databases and only verified by tumor tissue chips, not validated by in vivo and in vitro studies. Second, the role of *CD68* in tumor immune cell infiltration in pan-cancer was not verified by cell and animal experiments in this study. More studies focusing on the specific signaling pathways of *CD68* in pan-cancer need to be conducted in the future.

In summary, in this paper, we systematically discussed the aspects related to expression and mutant characteristics of *CD68* in pan-cancer. *CD68* levels were found to be elevated in many cancer types and showed significant prognostic value. These results, together with previous studies, demonstrate that *CD68* in the TME is a very promising biomarker in these tumors. In addition, *CD68* was found to be closely correlated with immune infiltrates in the TME and could a promising immunotherapy target that positively correlates with other checkpoint proteins in pan-cancer. Specific antibodies or inhibitors that neutralizing CD68 in the TME in there cancers might provide a new direction for tumor immunotherapy, especially targeting these immune cells that significantly associated with CD68 expression, such as B cells, CD4+ and CD8+ T cells, dendritic cells, macrophages, and neutrophils. Moreover, we explored various small molecules and predicted drugs that target *CD68* in pan-cancer. Multicenter clinical trials that focus on the efficacy, safety, or risk benefit ratio of small molecules and predicted drugs might benefit patients with elevated CD68 in these cancer tissues. Therefore, further research is needed to explore the therapeutic effects of these molecules for pan-cancer treatment through basic and clinical experiments.

## Supplementary Information

Supplementary Information 1.

Supplementary Figure 1.

Supplementary Figure 2.

Supplementary Figure 3.

Supplementary Figure 4.

Supplementary Figure 5.

Supplementary Figure 6.

Supplementary Figure 7.

Supplementary Table 1.

Supplementary Table 2.

Supplementary Table 3.

Supplementary Table 4.

## Data Availability

The datasets generated and analyzed during the current study are available from the corresponding author on reasonable request.
