# Supplementary material for: Role of CD68 in tumor immunity and prognosis prediction in pan-cancer
Source: Sci Rep. 2022 May 12;12:7844. doi: 10.1038/s41598-022-11503-2 (PMC9098459; doi:10.1038/s41598-022-11503-2)
Supplement: Supplementary file 1 — Supplementary Information 1. [file 41598_2022_11503_MOESM1_ESM.docx]

**Supplementary figures and tables legends**

**Supplement Figure 1. Survival analysis of *CD68* in pan-cancer from the TCGA database.** **Forest plot displaying the impact of high expression of *CD68* on DFI (A) and PFI (B) in pan-cancer using Cox regression model; Kaplan-Meier Method showed the prognostic value of *CD68* on DSS(C-I), DFI(J-M), and PFI(N-Q) in pan-cancer. The cut-off points are 56.67% (DSS in CESC), 72.64% (DSS in KIRC), 69.39% (DSS in GBM), 87.50% (DSS in KICH), 74.05% (DSS in LGG), 28.81% (DSS in THYM), 22.99% (DFI in CESC), 25.00% (DFI in CHOL), 12.97% (DFI in LIHC), 33.49% (DFI in STAD), 66.25% (PFI in GBM), 80.68% (PFI in KIRC), 36.54% (PFI in LGG), 37.98% (PFI in PRAD). *p< 0.05, **p < 0.01, ***p < 0.001. ACC: Adrenocortical carcinoma; BRCA: Breast invasive carcinoma; CESC: Cervical squamous cell carcinoma and endocervical adenocarcinoma; CHOL: Cholangiocarcinoma; COAD: Colon adenocarcinoma; ESCA: Esophageal carcinoma; GBM: Glioblastoma; HNSCC: Head and neck squamous cell carcinomas; KICH: Kidney chromophobe; KIRC: Kidney renal clear cell carcinoma; KIRP: Kidney renal papillary cell carcinoma; LAML: Acute myeloid leukemia; LGG: Low grade glioma; LIHC: Liver hepatocellular carcinoma; LUSC: Lung squamous cell carcinoma; OV: Ovarian Cancer; PAAD: Pancreatic adenocarcinoma; PCPG: Pheochromocytoma, and paraganglioma; PRAD: Prostate adenocarcinoma; READ: Rectum adenocarcinoma; SARC: Sarcoma; SKCM: Skin cutaneous melanoma; STAD: Stomach adenocarcinoma; TGCT: Testicular germ cell tumors; THCA: Thyroid carcinoma; THYM: Thymoma; ; UCS: Uterine carcinosarcoma; UVM: Uveal melanoma.**

**Supplement Figure 2. Correlation of *CD68* expression with immune infiltration level based on CIBERSORT in pan-cancer.**

**Supplement Figure 3. The correlation between *CD68* with the stromal score in pan-cancer.**

**Supplement Figure 4. The correlation between *CD68* with the immune score in pan-cancer.**

**Supplement Figure 5. The correlation between *CD68* with estimate score in pan-cancer.**

**Supplement Figure 6. The prognostic value of combination of *CD68* expression and MMR markers (A) and PD-1 (B).**

**Supplement Figure 7. The correlation between *CD68* expression and predicted drug response based on CellMiner in pan-cancer. *p< 0.05, **p < 0.01, ***p < 0.001.**

**Supplement Table 1. Relationship between neoantigen counts and *CD68* expression in pan-cancer**

**Supplement Table 2. Top 5 pathways based on GSEA of KEGG and HALLMARK terms of *CD68*.**

**Supplement Table 3. Top 20 predicted drugs’response correlated with *CD68* expression from the CTRP database**

**Supplement Table 4. Top 20 predicted drugs’response correlated with *CD68* expression from the GDSC database**
