# Supplementary figures and images for: Role of CD68 in tumor immunity and prognosis prediction in pan-cancer
Source: Sci Rep. 2022 May 12;12:7844. doi: 10.1038/s41598-022-11503-2 (PMC9098459; doi:10.1038/s41598-022-11503-2)

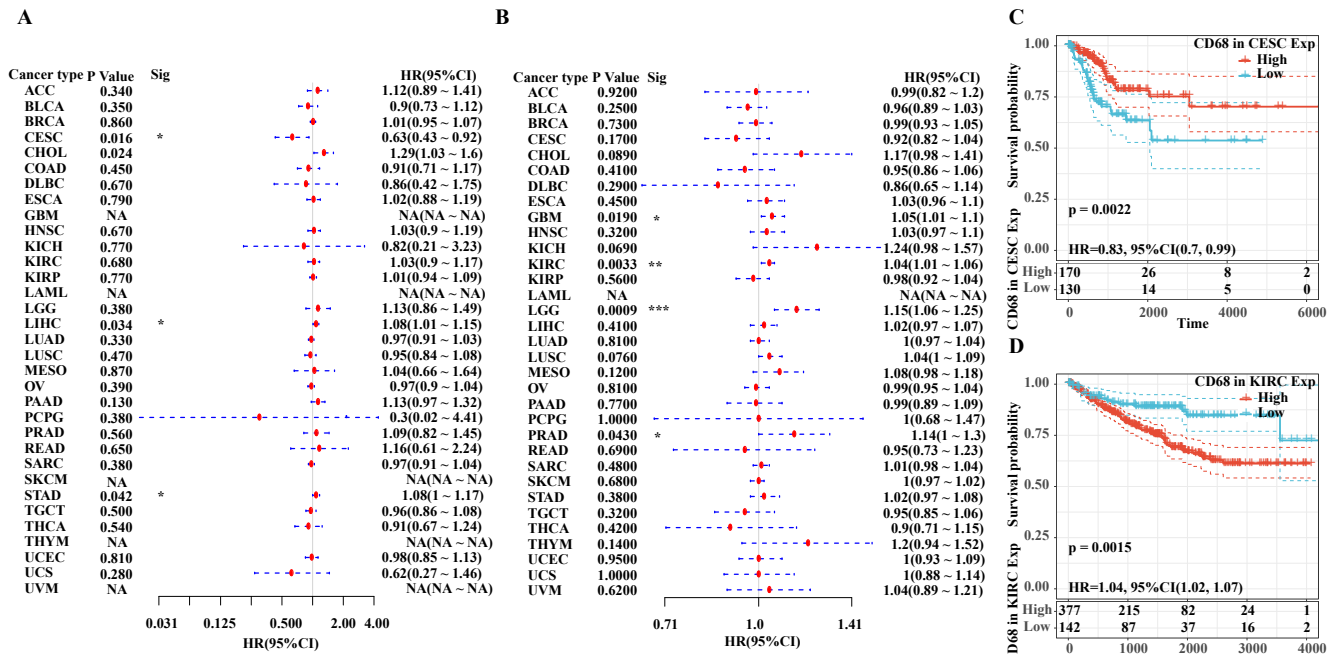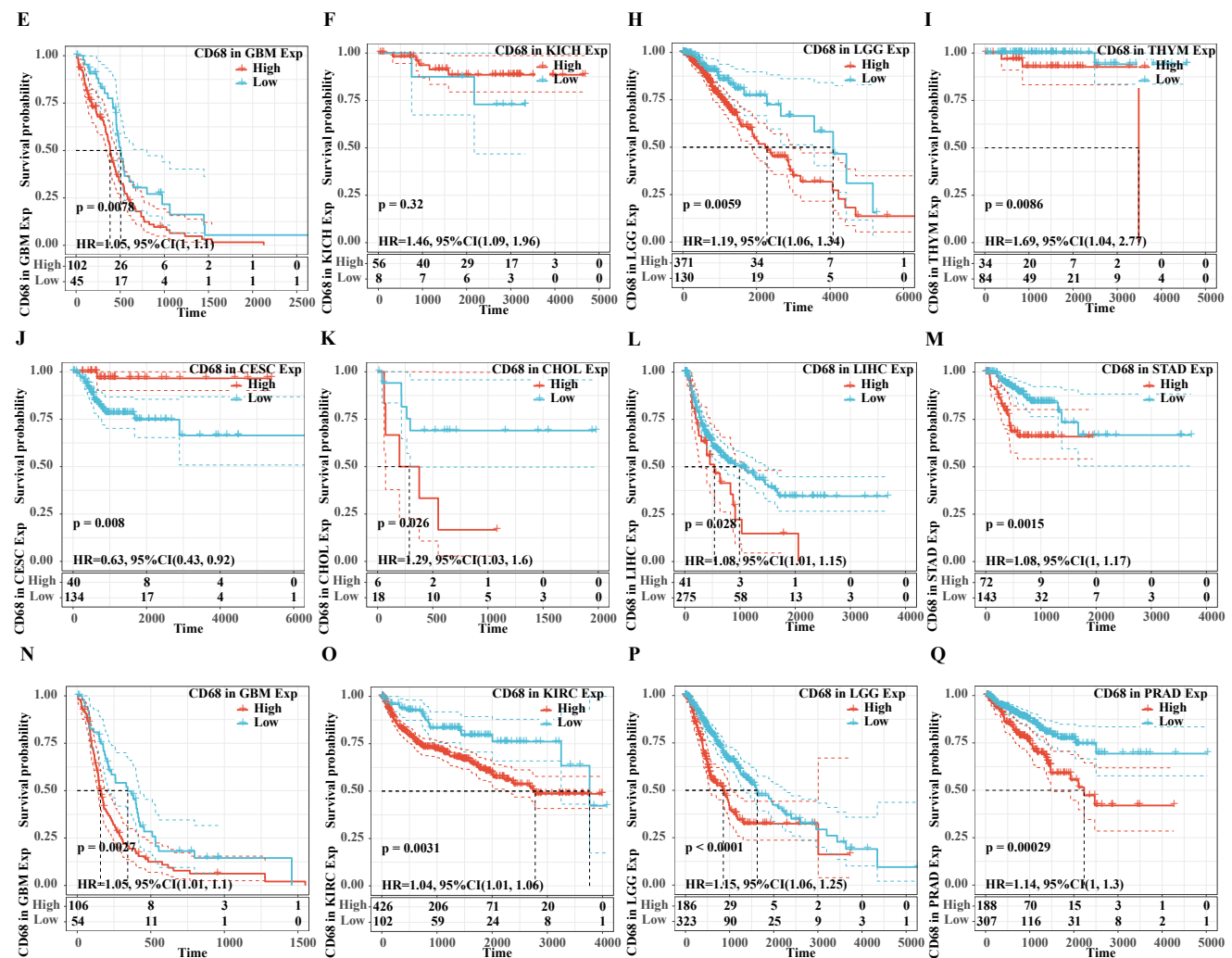

Supplement: Supplementary file 2 — Supplementary Figure 1. [file 41598_2022_11503_MOESM2_ESM.pdf]

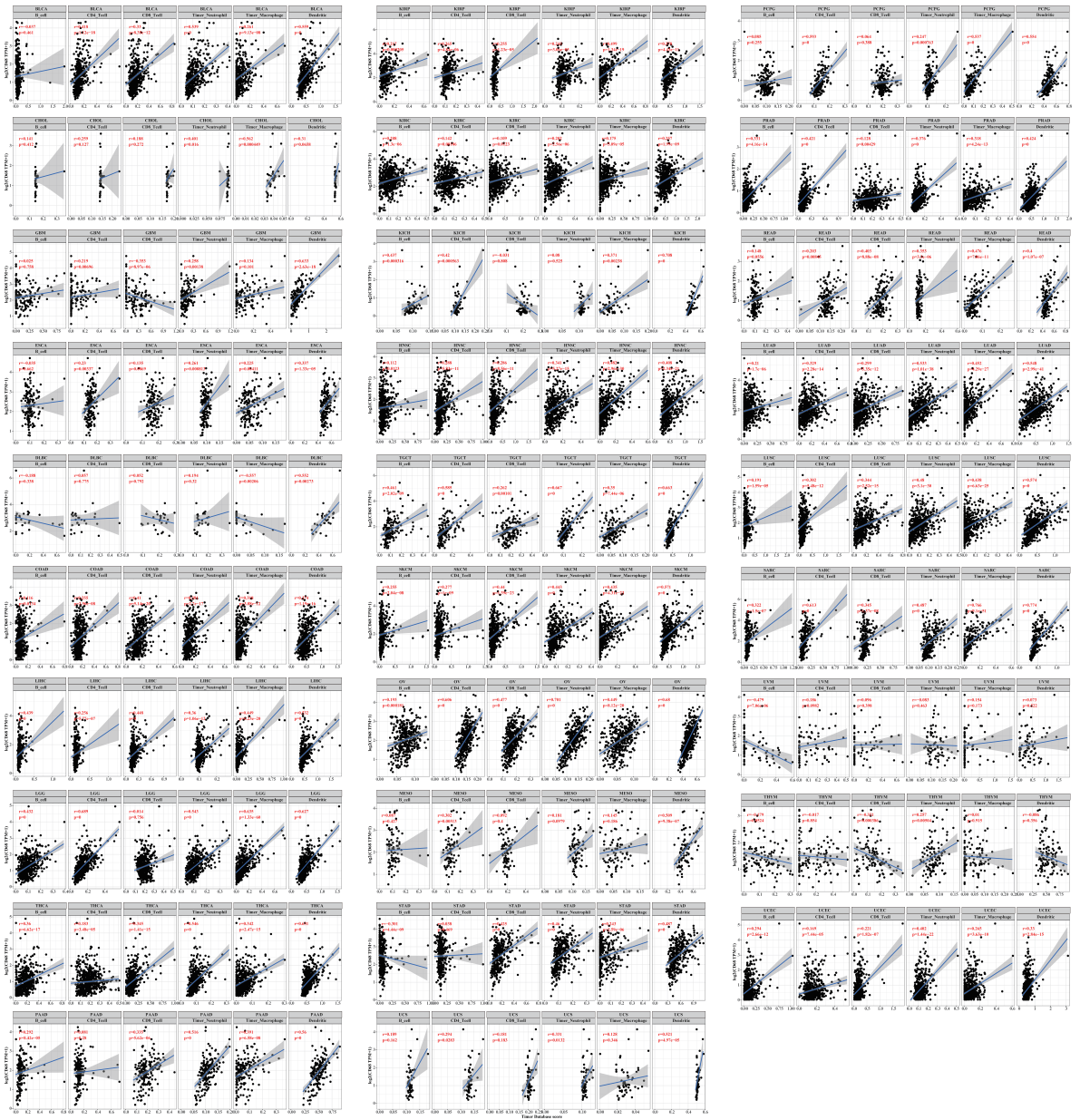

Supplement: Supplementary file 3 — Supplementary Figure 2. [file 41598_2022_11503_MOESM3_ESM.pdf]

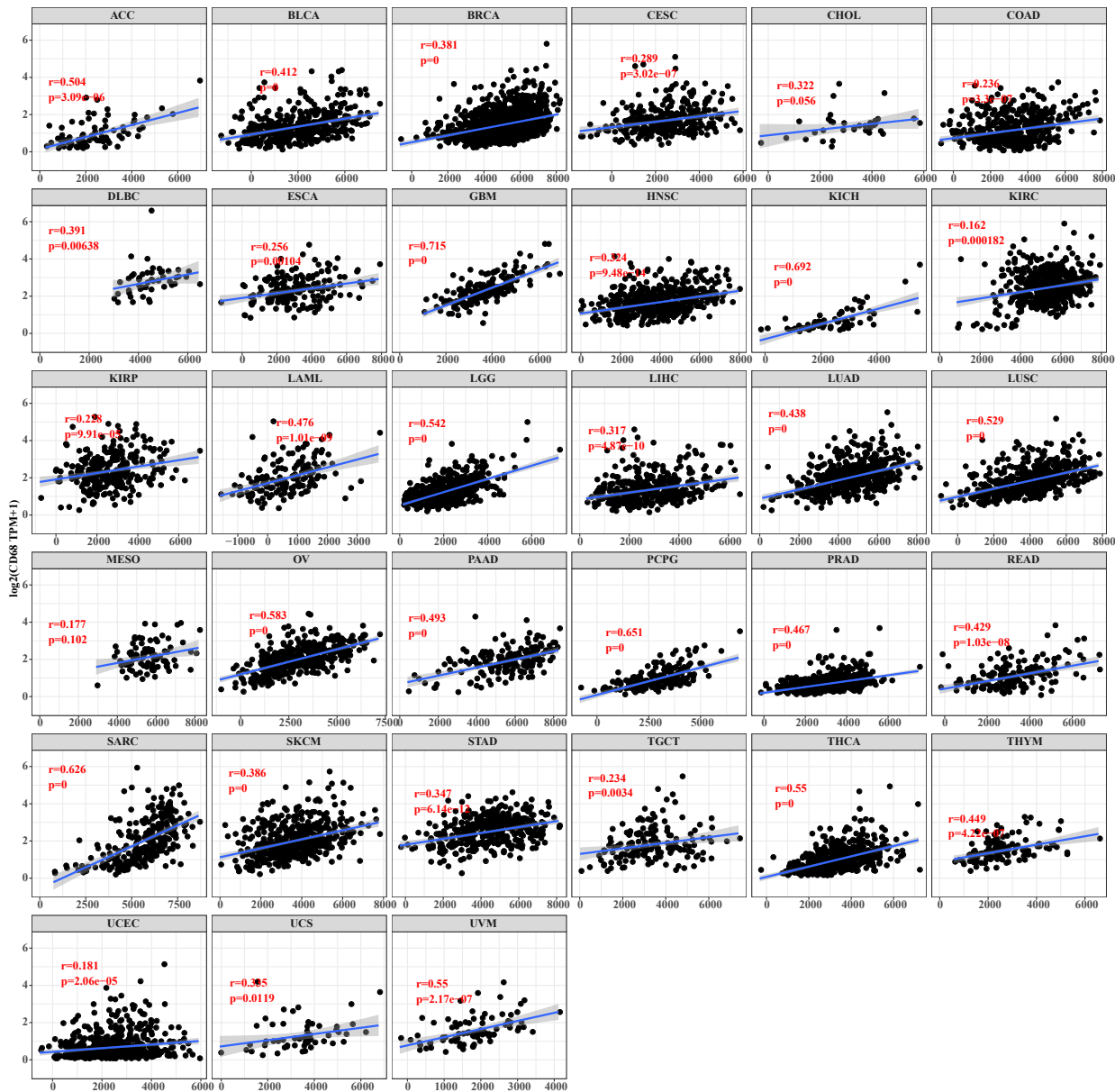

Supplement: Supplementary file 4 — Supplementary Figure 3. [file 41598_2022_11503_MOESM4_ESM.pdf]

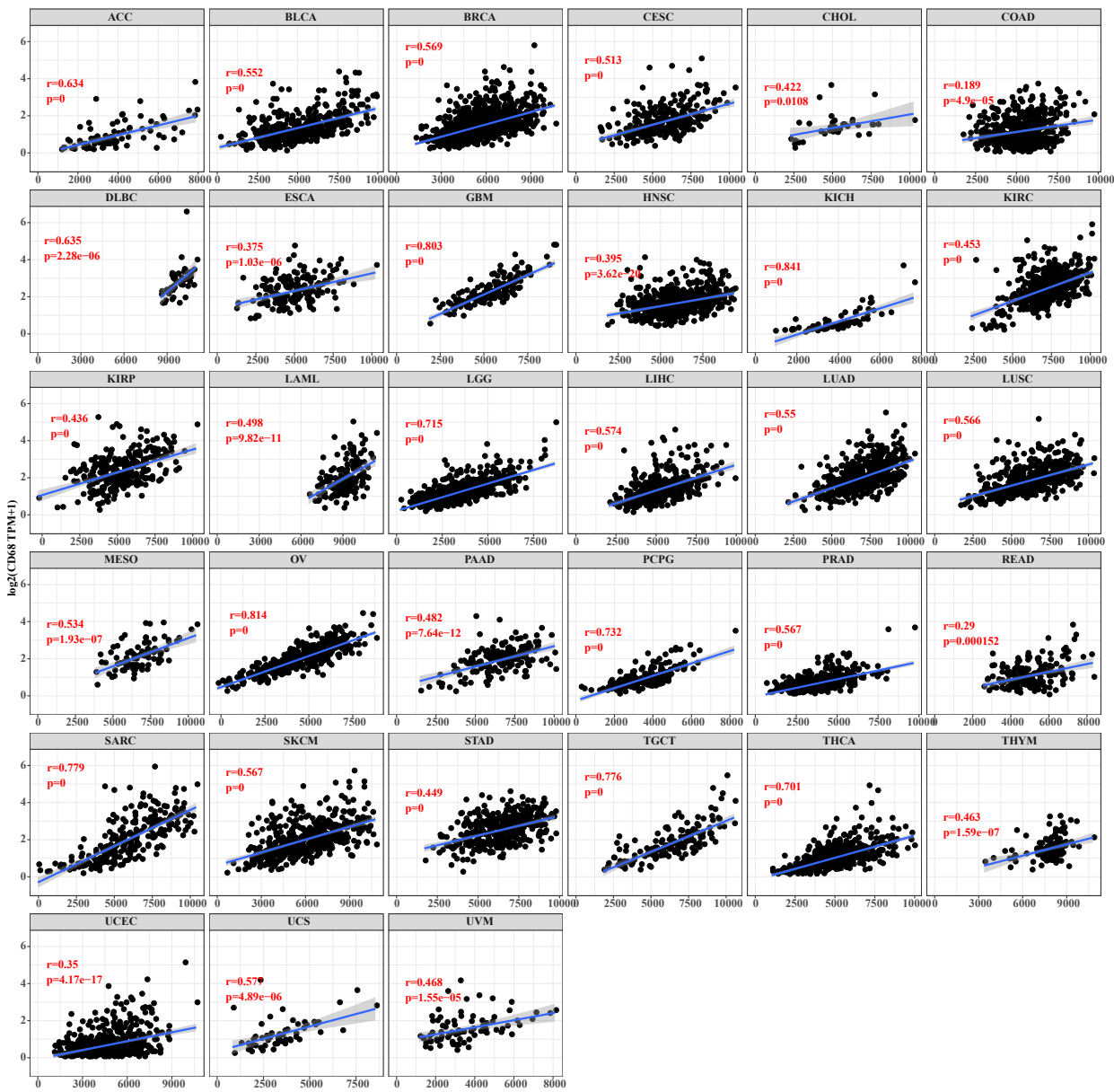

Supplement: Supplementary file 5 — Supplementary Figure 4. [file 41598_2022_11503_MOESM5_ESM.pdf]

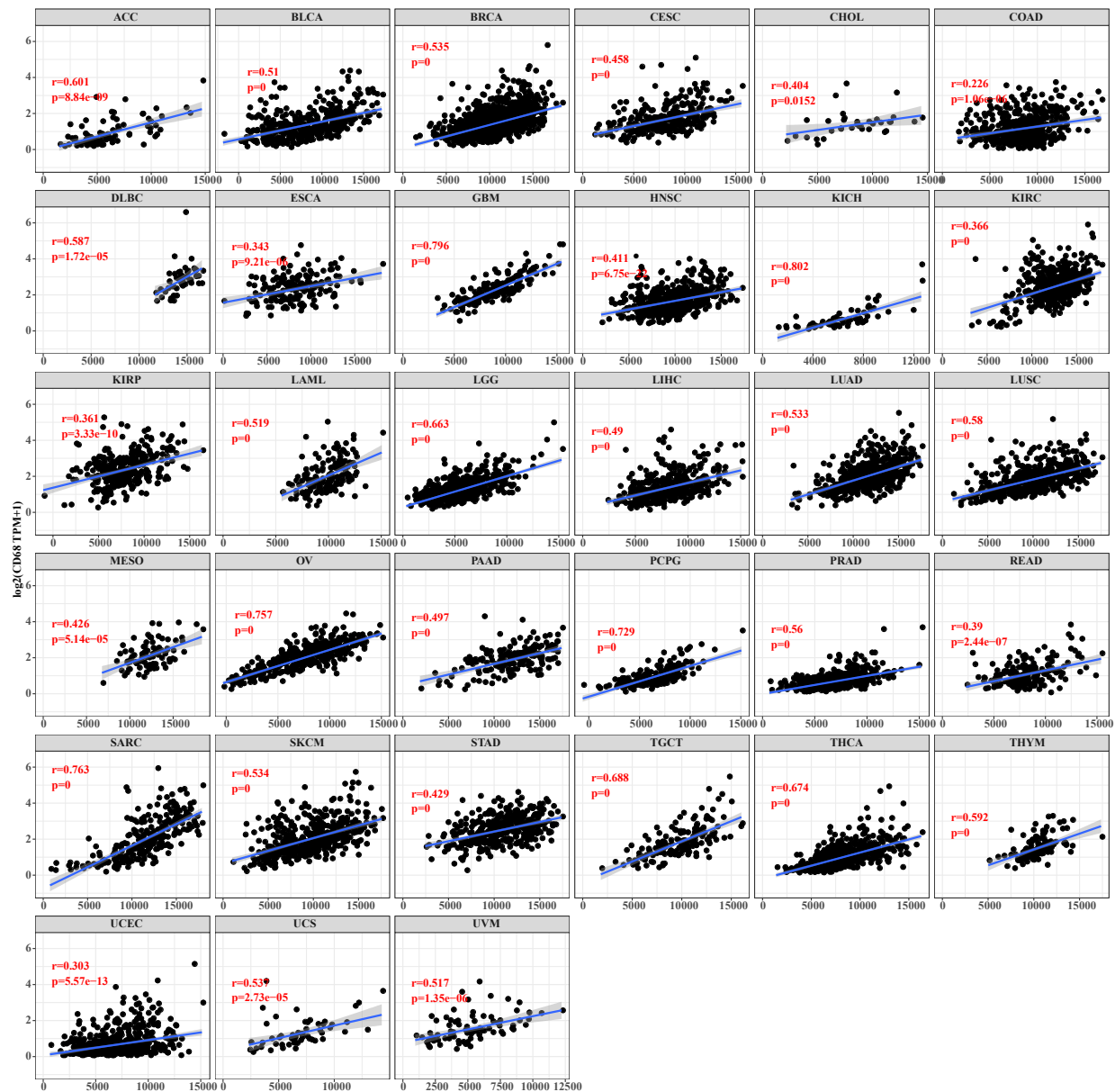

Supplement: Supplementary file 6 — Supplementary Figure 5. [file 41598_2022_11503_MOESM6_ESM.pdf]

**A**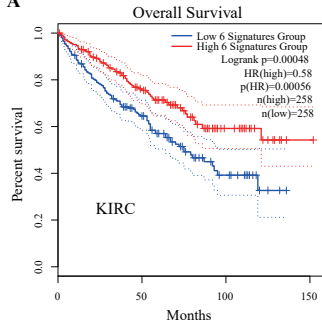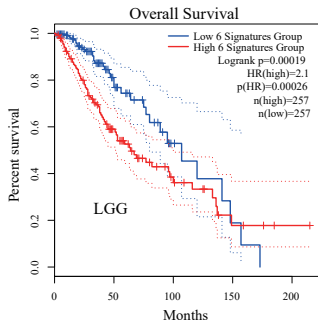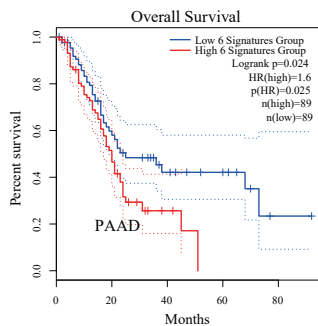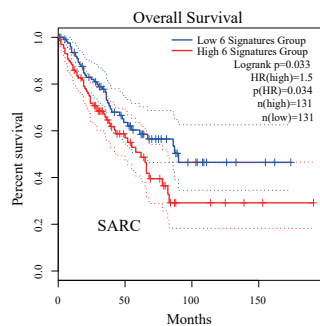**B**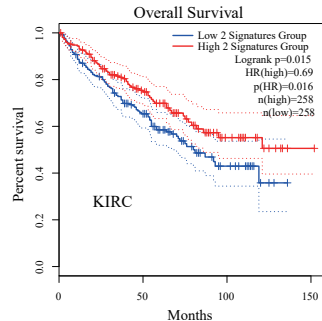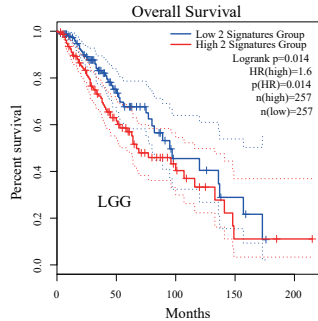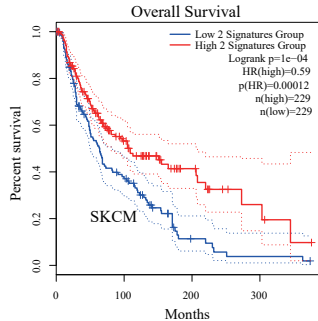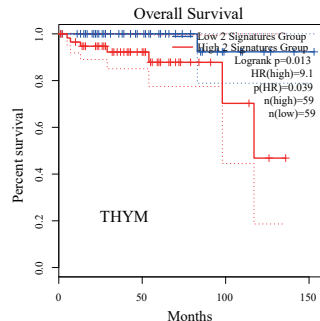

Supplement: Supplementary file 7 — Supplementary Figure 6. [file 41598_2022_11503_MOESM7_ESM.pdf]

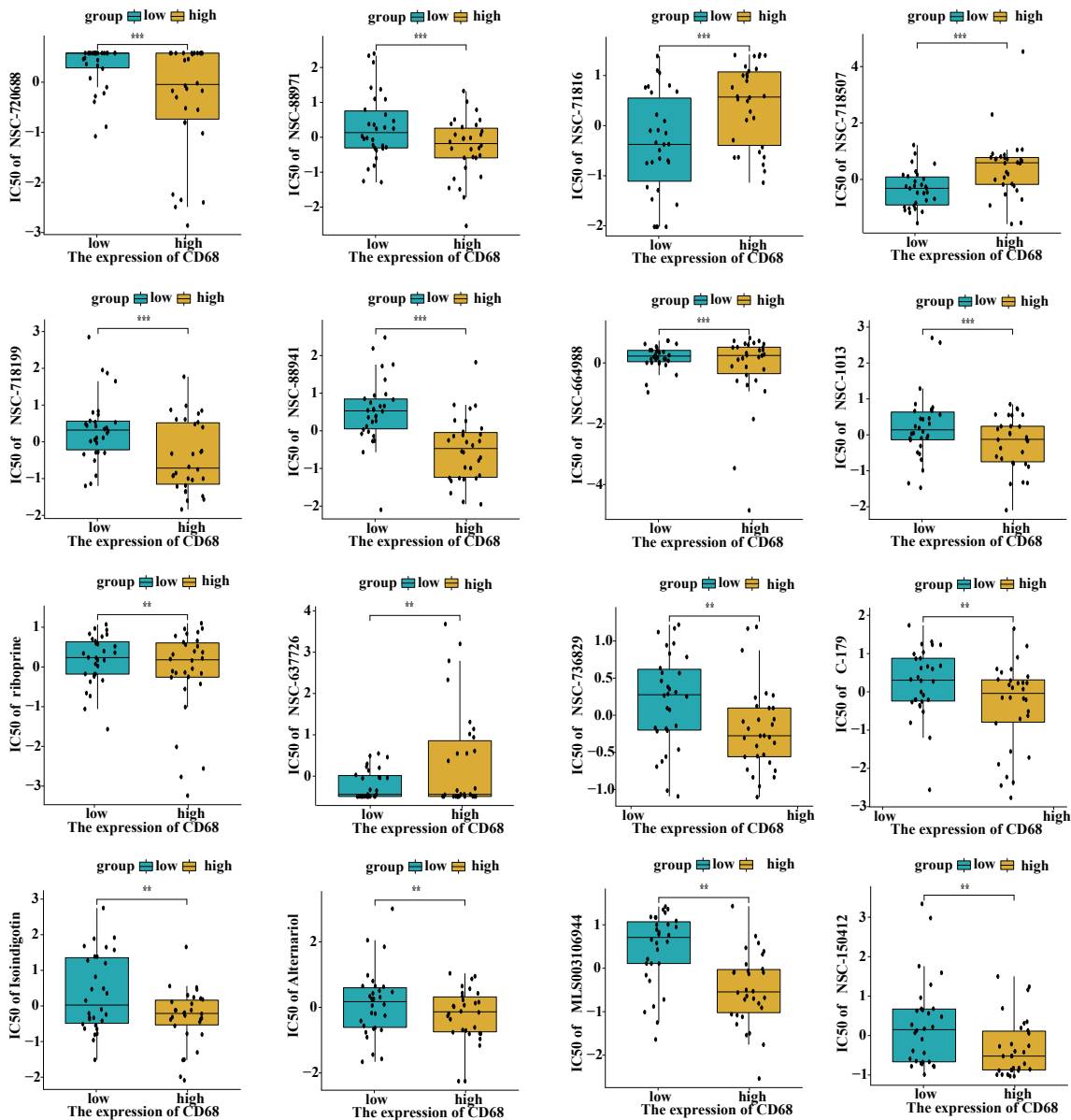

Supplement: Supplementary file 8 — Supplementary Figure 7. [file 41598_2022_11503_MOESM8_ESM.pdf]
